# Ideal Photothermal Materials Based on Ge Subwavelength Structure

**DOI:** 10.3390/molecules29215008

**Published:** 2024-10-23

**Authors:** Jingjun Wu, Kaixuan Wang, Cong Wei, Jun Ma, Hongbo Xu, Wanguo Zheng, Rihong Zhu

**Affiliations:** 1 School of Electronic and Optical Engineering, Nanjing University of Science and Technology, Nanjing 210094, China; jingjunwu163@163.com (J.W.); wangkaixuan20@gscaep.ac.cn (K.W.); weicong@njust.edu.cn (C.W.); group_ye@163.com (W.Z.); zhurihong@njust.edu.cn (R.Z.); 2 MIIT Key Laboratory of Critical Materials Technology for New Energy Conversion and Storage, School of Chemistry and Chemical Engineering, Harbin Institute of Technology, Harbin 150001, China

**Keywords:** photothermal absorption, subwavelength structure, RIE self-masking technique

## Abstract

Photothermal materials often prioritize solar absorption while neglecting thermal radiation losses, which diminishes thermal radiation conversion efficiency. This study addresses this gap by introducing a germanium (Ge) subwavelength structure (SWS) designed to optimize both solar absorption and infrared emissivity. Using a self-masked reactive ion etching (RIE) technique, we achieved a peak absorption of 98.8% within the 300 nm to 1800 nm range, with an infrared emissivity as low as 0.32. Under solar illumination of 1000 W/m^2^, the structure’s temperature increased by 50 °C, generating a heating power of 800 W/m^2^. Additionally, it demonstrated good mechanical and thermal stability at high temperatures and possessed a hydrophobic angle of 132°, ensuring effective self-cleaning. These characteristics make the Ge SWS suitable for application in solar panels, displays, sensors, and other optoelectronic devices.

## 1. Introduction

The global energy crisis remains a pressing issue, prompting a significant shift toward renewable energy sources, particularly solar energy, due to its abundance and cost-effectiveness [1,2,3]. The annual solar energy reaching the Earth’s surface is approximately 10^4^ times greater than current global energy consumption, highlighting its immense potential as a critical energy source [4]. Solar energy can be harnessed in various ways, such as electricity generation through photovoltaic systems, chemical energy storage in fuels like hydrogen, and thermal energy conversion via photothermal systems [5,6,7].

Among these methods, photothermal conversion, which utilizes a broader spectrum of sunlight and achieves higher energy conversion efficiencies, has garnered increasing attention [8]. Its diverse application in solar power generation are essential for maximizing solar energy utilization [9]. Conventional solar thermal systems typically employ photothermal materials to enhance solar energy absorption and conversion efficiency [10]. These materials include carbon-based nanomaterials, organic polymer nanoparticles, noble metal nanostructures, and semiconductor materials [11].

Carbon allotropes, such as carbon dots [12,13], nanodiamond [14,15], fullerene [16,17], graphite [18,19], carbon nanotubes (CNTs) [20,21], graphene [22] and their derivatives [23,24], are particularly well suited as photothermal materials due to their remarkable chemical stability, broad-spectrum light absorption, lightweight nature, and cost-effectiveness [25]. Their light-to-heat conversion depends on the excitation of loosely held π electrons and their subsequent relaxation to their ground state. Carbon-based composites exhibit a wide range of light absorption and excellent thermal conductivity [26]. This broad absorption capacity, ranging from ultraviolet to near-infrared light, optimizes the efficiency of solar energy utilization [27,28]. Additionally, they demonstrate high photon-to-heat conversion efficiency, thereby enhancing the performance of solar thermal systems in various applications, such as water heating and industrial processes. The preparation process for carbon materials is complex and costly, and the processability and compatibility with other materials are poor when fabricating thin films.

Polymers exhibit exceptional processability and compatibility, which has garnered significant attention. Recently, conjugated organic polymers, such as polythiophene (P.T.) [29], polypyrrole (PPy) [30], polyaniline (PAn), and polydopamine (PDA) [31], have emerged as promising photothermal nanomaterials. These polymers offer versatile molecular designs, strong near-infrared (NIR) light absorption, superior light-to-heat conversion efficiency, and excellent compatibility with biological systems [32]. Their absorption properties in the visible and NIR ranges are attributed to the nonradiative relaxation of delocalized π electrons, similar to those of carbon-based nanomaterials. Noble metal nanostructures, such as Au [33,34], Ag [35,36], Pd [37,38], Al [39,40], Cu [41,42], and Ge [43,44], also exhibit appealing photothermal conversion capabilities due to their localized surface plasmon resonances [45]. The frequency of these resonances can be finely tuned by adjusting the material composition, particle size, shape, and surrounding environment [46,47]. Noble metal ions and conductive polymers are costly and are not conducive to large-scale applications.

Semiconductor materials, including oxides, chalcogenides, nitrides, and silicon [48,49,50], are also highly effective for photothermal conversion due to their affordability, ease of fabrication, and low toxicity. Compared to organic photothermal materials, semiconductors exhibit superior resistance to photodegradation and photostability [51]. They also provide adjustable absorption wavelengths and high attenuation coefficients in the NIR range. The photothermal conversion efficiency of semiconductors is primarily determined by their bandgaps, which regulate the generation of free charge carriers and the subsequent heat production through the nonradiative recombination of electron–hole pairs. Titanium trioxide (Ti_2_O_3_), in particular, stands out for its exceptional photothermal conversion properties, with a bandgap energy of around 0.1 eV, allowing it to absorb energy across the entire solar spectrum [52]. Reducing the size of bulk Ti_2_O_3_ to the nanoscale enhances its light-scattering properties, thereby increasing its light absorption capacity [53]. Ti_2_O_3_ nanoparticles, with an ultrasmall bandgap of approximately 0.1 eV, have achieved nearly 100% internal and approximately 92% external solar-thermal conversion efficiency [54].

Despite these advances, challenges remain with existing photothermal materials. The optical properties of carbon-based nanomaterials are influenced by structural factors such as size, shape, doping, and layer number, which are closely linked to the fabrication process, making it difficult to produce materials that meet performance requirements [55]. Many organic light-absorbing materials suffer from instability due to ageing effects [56]. For metal nanoparticles, photothermal efficiency is influenced by particle assembly states (e.g., particle number, spacing and structure), necessitating careful consideration of particle arrangement and design [57]. Additionally, the fabrication of these materials can be complex and costly, limiting their widespread application. Black Ti_2_O_3_ particles are prone to aggregation [58], and their structural and oxidation properties require further investigation [59]. Furthermore, α-Ti_2_O_3_ can only be synthesized by reducing TiO_2_, which complicates its practical application [60]. Ideal photothermal materials must meet specific criteria, including a vast absorption capacity covering the entire solar spectral range (250–2500 nm), low infrared emissivity within the 2.5–25 µm range, to maximize conversion efficiency, and cost-effective scalability using abundant elements [61].

To address these limitations, this study introduces an innovative Ge SWS designed to optimize photothermal conversion efficiency by balancing high solar absorption with low infrared emissivity, thereby facilitating the development of ideal photothermal materials. This approach represents a significant improvement over traditional photothermal materials, which often neglect the effects of thermal radiation. The Ge SWS, fabricated using self-masked reactive ion etching, offers flexibility and tunability to adapt to various radiation and thermal conditions. Its exceptional mechanical and thermal stability at elevated temperatures, along with its self-cleaning properties, enhance its practical utility. Consequently, this research presents a promising strategy for improving the efficiency and durability of photothermal systems, with potential application in solar panels, displays, sensors, and other optoelectronic devices.

## 2. Results and Discussion

### 2.1. Generation of SWS

The self-masking effect is pivotal in the RIE process for generating the Ge SWS [62,63]. Due to the high volatility of SF_6_ ionization products, the formation of the self-mask primarily depends on the ionization products of CHF_3_, such as CHF*_x_, F*, F^−^, and CHF*_3_. Among these, the non-volatile fluorocarbon polymer C_x_H_y_F_z_ is particularly significant [64]. As shown in Equation (1), the combination of multiple CHF_3_ radicals and multiple electrons results in the formation of C_x_H_y_F_z_ polymers. These non-volatile polymers serve as masks during the RIE process, creating a competitive interaction between the etching and deposition of the C_x_H_y_F_z_ polymers [65]. When the etching rate of the C_x_H_y_F_z_ polymers exceeds their deposition rate, isotropic etching occurs, resulting in a uniformly etched substrate surface without the formation of SWS.

On the other hand, if the deposition rate of the C_x_H_y_F_z_ polymers significantly surpasses their etching rate, the entire surface becomes coated with the polymer, preventing the formation of well-defined nanostructures. The etching of Ge primarily relies on the chemical reactions involving fluorine ions (F*), as shown in Equation (2). These ions are mainly generated from the ionization of CHF_3_ and SF_6_ (SF_x_, F, F^−^, and SF*_6_). While SF_6_ enables rapid etching of the Ge substrate, it is an electronegative gas that consumes many electrons upon ionization, reducing the electron density in the discharge space. Helium is introduced to mitigate this effect and stabilize plasma discharge. Due to its lower mass, helium minimizes physical bombardment, thereby reducing damage to the substrate. By carefully adjusting the etching parameters—such as the gas flow ratios of SF_6_, CHF_3_ and He, the R.F. power, chamber pressure and sample stage temperature—the formation of C_x_H_y_F_z_ polymers can be modulated, maintaining a dynamic balance in the Ge etching rate and facilitating the creation of SWS.

**Figure 1:**
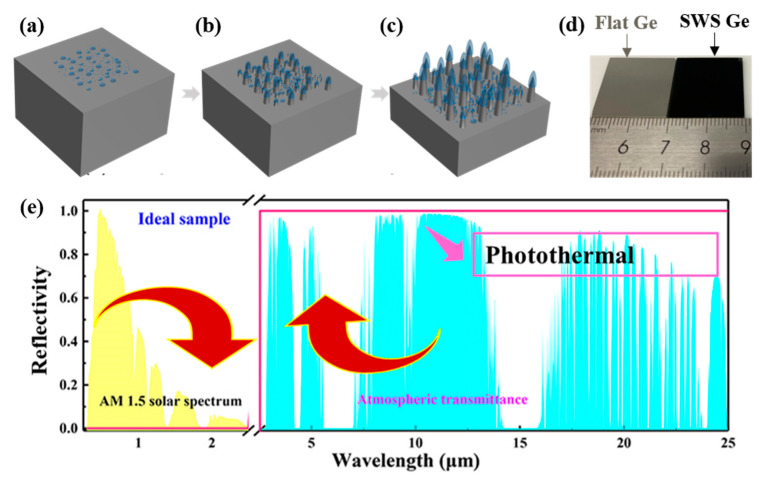
Generation of Ge SWS and photothermal effects. (**a**) Deposition of fluorocarbon polymer. (**b**) Formation of rough surfaces and partial nanostructures. (**c**) Generation of SWS. (**d**) Photograph of flat and SWS Ge. (**e**) Photograph of the ideal photothermal sample.

Figure 1a illustrates that C_x_H_y_F_z_ polymers randomly form on the substrate surface during the RIE process under suitable plasma conditions, despite the competition between etching and deposition. These polymers are etched by ion bombardment, while new polymers continuously form throughout the process [66]. Figure 1b shows that the C_x_H_y_F_z_ polymers remain on the surface during etching, resulting in the development of crests and valleys: the areas protected by the polymers become crests, while exposed areas become valleys. Over time, this effect amplifies, increasing the contrast between the crests and valleys.

Additionally, the deposition of C_x_H_y_F_z_ polymers is influenced by the surface roughness. Peaks on the surface accumulate more polymers than valleys. C_x_H_y_F_z_ polymer islands tend to form on the tips of nanopillars, further promoting polymer accumulation in those regions. As a result, initial nanostructures progressively enlarge, and the formed SWS grows more pronounced as the etching process continues. Figure 1c demonstrates that nanostructures of varying heights are produced across the sample surface due to the random timing and coverage of the mask formation. During the etching process, C_x_H_y_F_z_ polymers deposit on the sidewalls of the Ge SWS, providing additional protection and enhancing the formation of conical SWS, thereby significantly improving the vertical etching selectivity of Ge.

mCHF*_3_ + ne^−^→ C_x_H_y_F_z_ (1)

4F* + Ge → GeF_4_ (gas) (2)

Figure 1d displays images of planar Ge and Ge with SWS under sunlight. The photos reveal that planar Ge exhibits strong reflection, while Ge with SWS reflects almost none, appearing nearly black. This observation demonstrates the powerful absorption effect of our SWS. Figure 1e presents the ideal spectral curve of photothermal materials. The figure shows that the absorption in the solar spectral band (from 0.25 μm to 2.5 μm) is 1, while the infrared emission spectral band (from 2.5 μm to 25 μm) exhibits an infrared reflectance of 100% and an emissivity of 0. At this point, the surface photothermal conversion efficiency of the sample is maximized, achieving the maximum surface heating.

### 2.2. Effects of Process Parameters on SWS Formation

**Figure 2:**
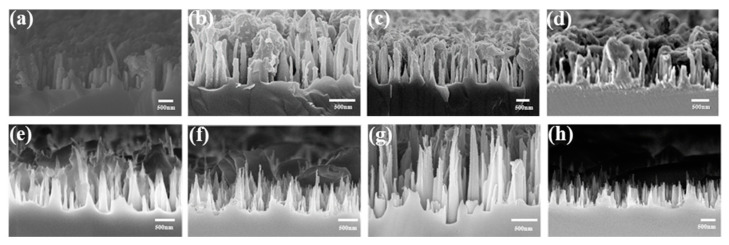
SEM images of Ge SWS under various conditions in Table 1: (**a**) Sample a, (**b**) Sample b, (**c**) Sample c, (**d**) Sample d, (**e**) Sample e, (**f**) Sample f, (**g**) Sample g, (**h**) Sample h.

The ratio of CHF_3_ gas significantly influenced the trials, as it was the primary source of C_x_H_y_F_z_ polymer formation in the self-masking process. This study explored various gas ratios, conducting experiments with different CHF_3_ concentrations, as detailed in Table 1 (Samples a–d). The corresponding SEM images are presented sequentially in Figure 2a–d. The height of the Ge SWS initially increased and then decreased with rising fluorocarbon ratios, with average measured heights of 600 nm, 900 nm, 800 nm and 400 nm and base widths ranging from 100 nm to 150 nm. As the CHF_3_ ratio increased, the spacing between Ge nanopillars also grew, accompanied by more C_x_H_y_F_z_ polymer deposition, as observed in Figure 2a,b. When the CHF_3_ concentration reached 45 SCCM and 50 SCCM, the balance between etching and deposition was disrupted, causing excessive accumulation of C_x_H_y_F_z_ polymers on the Ge SWS surface. This accumulation obstructed the etching gases from penetrating further, thereby reducing the depth-to-width ratio of the SWS, as shown in Figure 2c,d. Therefore, maintaining an optimal CHF_3_ ratio is critical for fabricating a high-quality Ge SWS.

The results indicate that a CHF_3_ concentration of 40 SCCM is optimal for producing a Ge SWS with a high depth-to-width ratio and minimal surface polymer deposition. It is important to note that fabricating a Ge SWS with a high aspect ratio makes it difficult for free radicals and ions to reach the bottom of the structure, which is necessary for the etching process. This complexity increases the challenge of removing etching by-products, potentially leading to a loading effect that can halt the etching process. In this study, the chamber pressure was reduced to 3 Pa to mitigate the loading effect, and the R.F. power was increased to 400 W. These adjustments facilitated the rapid removal of the by-product (GeF_4_) at the reduced pressure.

Additionally, the high power setting, which correlates with increased bias power, facilitates the removal of C_x_H_y_F_z_ polymers from the base through ion bombardment with high kinetic energy, reducing polymer accumulation at the base. Adjusting the CHF_3_ gas ratio is essential for fabricating a dense, high-aspect-ratio Ge SWS. Furthermore, optimizing other etching parameters effectively minimizes surface polymer content.

Etching time is another crucial factor influencing the depth-to-width ratio and surface C_x_H_y_F_z_ polymer content of the Ge SWS. Different etching durations were employed to prepare the SWS, as detailed in Table 1 (Samples e–h). The corresponding results are sequentially presented in Figure 2e–h. In Figure 2e,f, the Ge SWS exhibits average heights of approximately 400 nm and 600 nm, with depth-to-width ratios ranging from 3 to 4, and were covered with C_x_H_y_F_z_ polymers. A significant improvement in structural integrity was observed at an etching time of 45 min, where the average height reached 1000 nm, and the depth-to-width ratio peaked at 8:1, with minimal C_x_H_y_F_z_ polymers on the surface, as depicted in Figure 2g. At this etching duration, the deposition and etching achieved an optimal balance, resulting in a highly vertical Ge SWS with substantial depth-to-width ratios, dense peak spacing, and minimal surface polymer. This configuration enhanced reflection suppression across visible and near-infrared wavelengths.

However, extending the etching time to 50 min decreased the nanostructure height to approximately 600 nm and reduced the depth-to-width ratio to around 4, as shown in Figure 2h. Prolonged etching increased the substrate surface temperature, which hindered C_x_H_y_F_z_ polymer deposition. Consequently, the etching rate surpassed the deposition rate, disrupting the balance and shifting the process toward isotropic etching. This resulted in lower depth-to-width ratios for the Ge SWS and minimal residual C_x_H_y_F_z_ polymers on the surface.

The analysis confirms that the conditions described for Sample g in Table 1 produced a uniform Ge SWS (as further demonstrated by a larger area SEM oblique image in Figure S2 in Supplementary Materials) with optimal depth-to-width ratios. The findings indicate that both the depth-to-width ratio and the height of the Ge SWS initially increased with longer etching times before subsequently decreasing. Meanwhile, the bottom diameter remained relatively constant at around 150 nm, and the spacing between the spike structures was stable, with a gradual reduction in surface polymer accumulation.

### 2.3. Optical Properties

**Figure 3:**
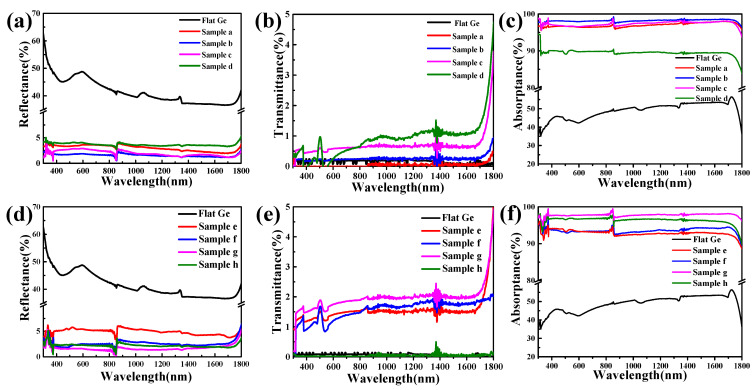
Optical properties of a Ge SWS under different conditions in Table 1. (**a**,**d**) The reflectance of Samples a–h. (**b**,**e**) The transmittance of Samples a–h. (**c**,**f**) The absorptance of Samples a–h.

We characterized the optical properties of the experimentally prepared Ge SWS. The average reflectance of flat Ge was approximately 45%, as shown in Figure 3a. Figure 3a–c correspond to the reflectance, transmittance, and absorptance measurements of Samples a–d in Table 1. As the depth-to-width ratios of the Ge SWS increased, the reflectance significantly decreased, reaching an average reflectance of less than 5% in the 300–1800 nm range, as depicted in Figure 3a. Initially, the reflectance of the Ge SWS decreased with increasing CHF_3_ proportions in the gas mixture but then rose again while the transmittance remained relatively unchanged. Notably, in Samples c and d, shown in Figure 3a, a higher proportion of CHF_3_ led to the deposition of a significant amount of C_x_H_y_F_z_ polymers. This excessive deposition, where the deposition rate exceeded the etching rate, reduced the depth-to-width ratio of the Ge nanospike structures, as illustrated in Figure 2c,d. Therefore, precise control of the CHF_3_ proportion in the gas mixture is critical for optimizing the etching process.

The Ge SWS prepared in this experiment achieved a minimum reflectance of 1.2% in the visible range. Figure 3d–f correspond to the reflectance, transmittance, and absorptance measurements of Samples e–h in Table 1. As shown in Figure 3d,e, the reflectance decreased with increasing etching time and then slightly increased, while transmittance remained between 0 and 2%, indicating almost no transmittance. Comparing the optimized Sample g with flat Ge, the average reflectance of the Ge nanostructure was approximately 2%, significantly lower than the 45% reflectance of flat Ge over the 300–1800 nm range.

Thus, the Ge SWS prepared after process optimization exhibited excellent broadband antireflective properties from the visible to the near-infrared (Vis-NIR) range, especially in the visible range, achieving a minimum reflectance of 1.2% with negligible transmittance, corresponding to an absorption rate of 98.8% as shown in Figure 3f. The optical characterization results highlight that the high aspect ratio of the SWS is crucial for attaining broadband antireflective performance. This finding suggests that taller SWS with the same bottom diameter as the Ge SWS would offer even better broadband antireflective performance. The low reflectance of the Ge nanocone surface in the visible to near-infrared range can be attributed to the small size and high depth-to-width ratio of the SWS, which gradually increases the effective refractive index from the air to the Ge substrate, thereby reducing reflections at the optical interfaces.

### 2.4. Radiant Heat Absorption

**Figure 4:**
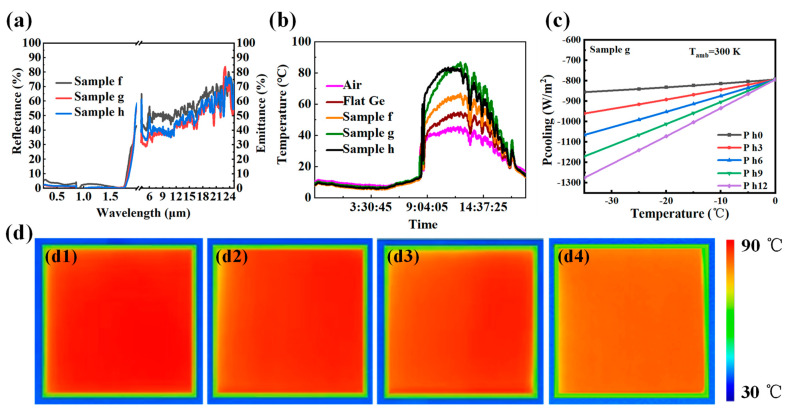
Radiant heat absorption of a Ge SWS in Table 1. (**a**) Reflectance and emittance spectra for Samples f, g, and h. (**b**) Outdoor temperature test curves for air, flat Ge and Samples f, g, and h. (**c**) Calculated heating power for Sample g. (**d**) Infrared thermal images of flat Ge (**d4**), Sample f (**d3**), Sample g (**d1**), and Sample h (**d2**).

Figure 4 presents the radiant heat absorption characteristics of a Ge SWS as outlined in Table 1. Figure 4a illustrates the reflection spectra for the solar and infrared radiation bands of Samples f, g, and h. Its reflectivity is extremely low, with a peak absorption rate of 98.8% across the 250 nm to 1800 nm range. The spectral integration calculation shows absorption rates of 97%, 98%, and 97% for Samples f, g, and h, respectively, over the 250 nm to 1800 nm range. The corresponding absorption efficiencies over the broader 250 nm to 2500 nm range are 94%, 95%, and 94%, respectively. Using blackbody radiation and infrared spectrum integration, the average infrared emission of Samples g, h, and f in the range of 2.5 μm to 25 μm is calculated to be 0.32, 0.35, and 0.36, respectively, which aligns with the spectral requirements for solar heating. Figure 4b shows the results from an outdoor temperature measurement experiment conducted during summer in Harbin. The data indicate that the sample can be heated to 90 °C when the ambient temperature is 40 °C, resulting in a temperature increase of 50 °C. The heating power of the sample was calculated using the heat balance equation and was found to be 800 W/m^2^ (Figure 4c). Figure 4d provides infrared thermal imaging data, showing a temperature rise of 85 °C. The infrared thermal images in Figure 4d correspond to the points of maximum sample temperature as depicted in Figure 4b. The infrared thermal images of Sample g, Sample h, Sample f, and flat Ge are shown in Figure 4d1–d4, respectively.

### 2.5. Hydrophilicity Test

**Figure 5:**
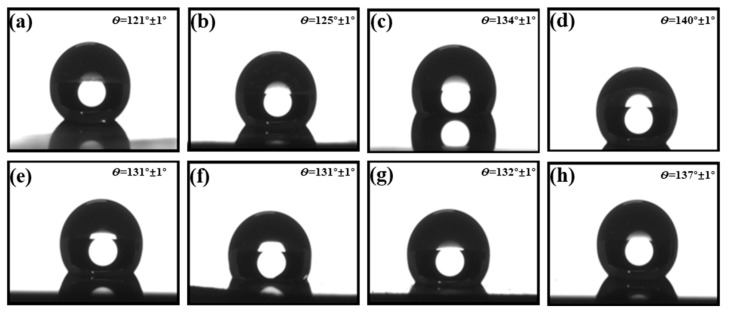
Hydrophilic properties of Ge SWS under different conditions in Table 1: (**a**) Sample a, (**b**) Sample b, (**c**) Sample c, (**d**) Sample d, (**e**) Sample e, (**f**) Sample f, (**g**) Sample g, (**h**) Sample h.

The prepared Ge SWS was experimentally tested for hydrophobicity, revealing a contact angle of 70° for flat Ge, as detailed in Figure S1 in Supplementary Materials. Figure 5a–d correspond to Figure 2a–d. As the CHF_3_ ratio increased, the hydrophobic contact angle rose from 121° to 140°. This increase is attributed to the intrinsic hydrophobic nature of the C_x_H_y_F_z_ polymers deposited on the surface of the Ge SWS, along with the enhanced hydrophobicity provided by the high depth-to-width ratios, which were approximately four. Therefore, both the high aspect ratio of the Ge SWS and the substantial coverage of the C_x_H_y_F_z_ polymers contributed to the observed improvement in hydrophobicity.

The hydrophobic contact angle measurements in Figure 5e–h correspond to Figure 2e–h. The overall contact angle ranged from 131° to 137°, higher than that of flat Ge. In these samples, the amount of fluorocarbon polymer on the surface gradually decreased. While the depth-to-width ratio of the Ge SWS initially increased and then decreased, the maximum contact angle of 137° was observed when the depth-to-width ratio was around eight. Despite the reduction in fluorocarbon polymer content, the hydrophobic contact angle remained stable between 131° and 137°, suggesting that SWS with a high depth-to-width ratio can maintain effective hydrophobicity even with a diminished surface polymer presence.

These enhanced hydrophobic properties have potential applications in solar panels, displays, and sensors. However, the presence of fluorocarbon polymer residues on the surface of the SWS may adversely affect the performance of these devices. Future work will focus on reducing or eliminating the C_x_H_y_F_z_ polymers while preserving the optical properties and hydrophobicity of the Ge SWS.

## 3. Materials and Methods

The Ge SWS was fabricated using fluorocarbon radical plasma etching in an RIE system. Before etching, the RIE chamber was cleaned for 20 min with an argon and oxygen plasma mixture to ensure consistent initial conditions for each etching process. First, the Ge wafers (crystal orientation <111>, thickness ~1.0 mm, dimensions 2.0 cm × 2.0 cm) were cleaned in an ultrasonic bath with acetone for 15 min. After washing, the wafers were dried using a nitrogen stream and immediately transferred to the RIE chamber. The gaseous reactants for the etching process included sulfur hexafluoride (SF_6_), trifluoromethane (CHF_3_), and helium (He). The radio frequency power was maintained at 400 W during etching, and the chamber pressure was constant at 3 Pa. The etching duration ranged from 35 to 50 min. The parameters are detailed in Table 1.

The surface morphology of the etched Ge SWS was characterized using a scanning electron microscope (SEM, ZEISS FESEM ULTRA 55, Oberkochen, Germany). Before imaging, the planar Ge substrate with the SWS was sputter-coated with a thin layer of gold to enhance imaging quality. A contact angle tester (KRUSS DSA30, Hamburg, Germany) was used to evaluate the hydrophilic or hydrophobic properties of the structures. Contact angle measurements were conducted by applying 30 μL of distilled water to the surface. The optical properties of the etched Ge wafers, including transmittance and reflectance, were examined using a UV–visible spectrophotometer (PerkinElmer lambda 950, Shelton, CT, USA), providing detailed insights into the optical behavior of the SWS surface.

## 4. Conclusions

This study successfully fabricated a Ge SWS using a self-masking RIE technique, demonstrating exceptional photothermal conversion efficiency. The optimized Ge SWS exhibited high solar absorption (a peak absorption of 98.8% across the 300–1800 nm range) and an infrared emissivity as low as 0.32, effectively balancing solar energy absorption with minimized thermal radiation loss. The structure also achieved a high depth-to-width ratio (~8:1) with good uniformity, resulting in excellent optical performance, with reflectance as low as 2.4% and radiant heating power of up to 800 W/m^2^ under sunlight. In addition to its impressive optical properties, the Ge SWS demonstrated robust hydrophobicity, with a contact angle of 132°, making it well suited for application in solar panels, displays, and sensors. The combination of high-performance photothermal absorption, antireflective efficiency, and hydrophobic properties makes the Ge SWS a promising candidate for various optoelectronic devices, contributing to advancements in solar energy utilization, CMOS technology, and spectroscopic applications. This work provides a scalable and cost-effective approach to enhancing the efficiency of photothermal conversion technologies.

## Figures and Tables

**Table 1:** Experimental conditions for the self-masking RIE process.

Sample	Etching Time (min)	Pressure (Pa)	Power (W)	Gas Ratio (CHF_3_:SF_6_:He (SCCM))
a	40	3	400	35/10/5
b	40	3	400	40/10/5
c	40	3	400	45/10/5
d	40	3	400	50/10/5
e	35	3	400	40/10/10
f	40	3	400	40/10/10
g	45	3	400	40/10/10
h	50	3	400	40/10/10

## Data Availability

The data that support the plots within this paper are available from the corresponding authors upon request.

## Supplementary Materials

The following supporting information can be downloaded at https://www.mdpi.com/article/10.3390/molecules29215008/s1. Figure S1: Hydrophobic contact angle test of Flat Ge. Figure S2: SEM image of Sample g in Table 1.
