# Supplementary material for: Ideal Photothermal Materials Based on Ge Subwavelength Structure
Source: Molecules. 2024 Oct 23;29(21):5008. doi: 10.3390/molecules29215008 (PMC11547708; doi:10.3390/molecules29215008)
Supplement: Supplementary file 1 [file molecules-29-05008-s001.zip › molecules-3266103-supplementary.pdf]

Ideal photothermal materials based on Ge subwavelength structure

Jingjun Wu [1,†], Kaixuan Wang [1,†], Cong Wei [1], Jun Ma [1,*], Hongbo Xu [2,], Wanguo Zheng [1], Rihong Zhu [1]**

[1] School of Electronic and Optical Engineering, Nanjing University of Science and Technology, Nanjing 210094, China.
[2] MIIT Key Laboratory of Critical Materials Technology for New Energy Conversion and Storage, School of Chemistry and Chemical Engineering, Harbin Institute of Technology, Harbin 150001, China.
* Correspondence: * majun@njust.edu.cn, **iamxhb@hit.edu.cn.
† These authors contributed equally to this work.

Part 1: extended data figures

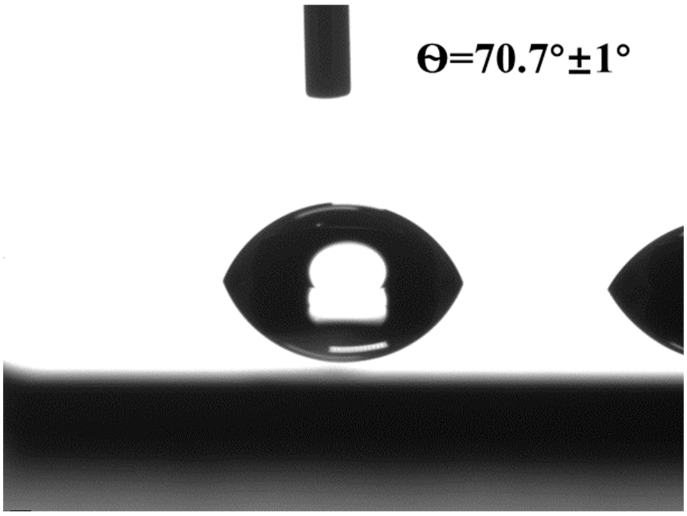

Figure S1 Hydrophobic contact angle test of Flat Ge.

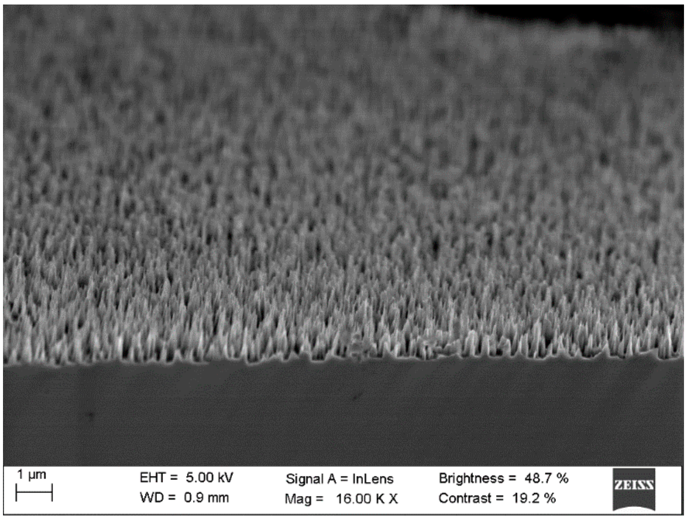

Figure S2 SEM image of Sample g in Table 1.
